# Improve sleep in critically ill patients: Study protocol for a randomized controlled trial for a multi-component intervention of environment control in the ICU

**DOI:** 10.1371/journal.pone.0286180

**Published:** 2023-05-25

**Authors:** Leyla Alegria, Pablo Brockmann, Paula Repetto, Douglas Leonard, Rodrigo Cadiz, Fabio Paredes, Idalid Rojas, Ana Moya, Vanessa Oviedo, Patricio García, Jan Bakker

**Affiliations:** 1 Intensive Care Medicine Department, School of Medicine, Pontificia Universidad Católica de Chile, Santiago, Chile; 2 School of Nursing, Pontificia Universidad Católica de Chile, Santiago, Chile; 3 Division of Pediatrics, Department of Pediatric Pulmonology, School of Medicine, Pontificia Universidad Católica de Chile, Santiago, Chile; 4 Pediatric Sleep Center, School of Medicine, Pontificia Universidad Católica de Chile, Santiago, Chile; 5 School of Psychology, Pontificia Universidad Católica de Chile, Santiago, Chile; 6 School of Design, Pontificia Universidad Católica de Chile, Santiago, Chile; 7 Faculty of Arts, Music Institute, Pontificia Universidad Católica de Chile, Santiago, Chile; 8 Department of Electrical Engineering, School of Engineering, Pontificia Universidad Católica de Chile, Santiago, Chile; 9 Faculty of Mathematics, Pontificia Universidad Católica de Chile, Santiago, Chile; 10 Department of Health Sciences, School of Kinesiology, School of Medicine, Pontificia Universidad Católica de Chile, Santiago, Chile; 11 Department of Intensive Care, Erasmus MC University Medical Center, Rotterdam, The Netherlands; 12 Department of Pulmonology and Critical Care, Columbia University Medical Center, New York, New York, United States of America; 13 NYU School of Medicine Langone, New York, New York, United States of America; Universidad de La Sabana, COLOMBIA

## Abstract

**Introduction:**

In critically ill patients, sleep and circadian rhythms are greatly altered. These disturbances have been associated with adverse consequences, including increased mortality. Factors associated with the ICU environment, such as exposure to inadequate light and noise levels during the day and night or inflexible schedules of daily care activities, have been described as playing an essential role in sleep disturbances. The main objective of this study is to evaluate the impact of the use of a multifaceted environmental control intervention in the ICU on the quantity and quality of sleep, delirium, and post-intensive care neuropsychological impairment in critically ill patients.

**Methods:**

This is a prospective, parallel-group, randomized trial in 56 critically ill patients once they are starting to recover from their acute illness. Patients will be randomized to receive a multifaceted intervention of environmental control in the ICU (dynamic light therapy, auditory masking, and rationalization of ICU nocturnal patient care activities) or standard care. The protocol will be applied from enrollment until ICU discharge. Baseline parameters, light and noise levels, polysomnography and actigraphy, daily oscillation of plasma concentrations of Melatonin and Cortisol, and questionnaires for the qualitative evaluation of sleep, will be assessed during the study. In addition, all patients will undergo standardized follow-up before hospital discharge and at 6 months to evaluate neuropsychological impairment.

**Discussion:**

This study is the first randomized clinical trial in critically ill patients to evaluate the effect of a multicomponent, non-pharmacological environmental control intervention on sleep improvement in ICU patients. The results will provide data about the potential synergistic effects of a combined multi-component environmental intervention in ICU on outcomes in the ICU and long term, and the mechanism of action.

**Trial registration:**

ClinicalTrials.gov, NCT. Registered on January 10, 2023. Last updated on 24 Jan 2023.

## Introduction

### Background

Sleep is a natural process that is critical for health and well-being, essential for rest, repair, and the survival of the individual. It is controlled by a circadian system that drives 24-h periodicity and a homeostatic system that ensures adequate amounts of sleep are obtained [1]. External factors such as the light/dark cycle can affect the circadian rhythm, interact with internal clocks by synchronizing their different phases, and, consequently affect normal sleep [1–4]. Sleep and circadian rhythms are markedly disturbed in intensive care unit (ICU) patients. These alterations occur in sleep continuity, sleep architecture, and regularity, as well as abnormal oscillations in melatonin, cortisol, and body temperature [3–8]. Sleep disruption is one of the most frequent complaints from ICU patients [5, 6, 8], and it is associated with several adverse consequences increasing the risk of adverse hospital outcomes, including mortality [6, 9–13]. Disrupted sleep can also result in anxiety, pain, self-reported feelings of depressed mood, anger, frustration, and tension in ICU patients [11, 14, 15]. Long-term, sleep deprivation significantly affects cognitive function. An association between delirium and sleep disturbance has been suggested, as prolonged sleep deprivation over several days can trigger perceptual distortions and hallucinations in healthy individuals, and all these effects may play a role in the occurrence of delirium among ICU patients [3–8, 12, 16]. Factors related to sleep deprivation in critically ill patients are varied and derive from the patient’s history, critical illness, and therapeutic and care interventions. Factors such as the type, severity, and pathophysiology of the critical illness, pain (due to procedures or critical illness) and stress/anxiety are factors that are related to sleep deprivation in critically ill patients. [6]. Similarly, several commonly used ICU medications, including vasopressors, antibiotics, sedatives, opioids, and analgesics, may have profound effects on sleep quantity and quality [6, 9, 16–18]. Although these factors related to the patient’s condition play a role in sleep disruption, they are difficult to modify. However, factors associated with the ICU environment have also been reported to have a major role in sleep disturbance (e.g., lighting, noise, and patient care activities), but they can be modified by changes in ICU design and culture [19].

The ICU environment is disruptive and does not favor rest. A night in a typical ICU has been described as a “chorus of alarms, voices, and telephone rings, direct and indirect light pollution, and interruptions from unfamiliar care providers” [20]. In this context, several environmental factors that could contribute to altering a patient´s sleep can be identified. Surveys carried out on patients and quantitative measures indicate that the main environmental factors that produce alterations in sleep are light, noise, and interruptions due to care activities [21–24]. Descriptive studies have defined and expanded our understanding of the relationship between environmental problems and serious sleep disturbances in the ICU [25–39]. Consequently, several studies have evaluated interventions targeting sleep optimization in the ICU, including non-pharmacologic and pharmacologic interventions [21, 30, 40–51]. As for pharmacological therapy, this should be for short periods, with continuous re-evaluation of the need due to adverse effects. [3–8]. Non-pharmacologic interventions should be the main focus of future efforts, particularly those directed at improving the ICU environment.

Light is a major cause of sleep disruption in the ICU setting [52–54]. The light-dark cycle is the most powerful entraining factor in the human circadian rhythm and the sleep-wake cycle [1, 2]. The ability of light to modulate cortical activity and circadian rhythm is defined, by the duration, intensity (lux [lx]), and wavelength (nm) of the lighted stimulus [55, 56]. Warmer color temperatures (ranging from < 2700K to 3500K) represent daylight hours when the sun is rising or setting, and when people should be waking up or falling asleep [55]. Endogenous melatonin secretion occurs in parallel with the light-dark cycle, with melatonin concentrations reaching low values during the daytime and high values at night [56, 57]. Therefore, the indoor lighting system should be dynamic, providing greater intensity and color temperatures during the morning, to support the awakening, decreasing to a more standard intensity later down, and finally, in the afternoon, reducing the intensity of light to promote melatonin secretion [55, 56]. In ICU, the primary purpose of lighting has been to support the treatment and monitoring of patients with severe diseases. Studies that have measured light in the ICU have generally documented lower than recommended levels during the day and at night [19], which may ultimately lead to sleep disturbances [3, 16, 58] and have a significant effect on the circadian pacemaker [56]. Because light is such an important factor in the regulation of sleep and wakefulness, modulating light exposure in the ICU could restore the temporal disorganization of circadian pacemakers. Indeed, light has been applied therapeutically as a treatment for sleep disorders in other patient populations [20, 59]. Although no study has directly evaluated the effect of an enhanced lighting system on ICU sleep, a couple of studies have tested the impact of dynamic light as a single strategy on other related outcomes, such as patient satisfaction, delirium, and circadian rhythm. Results have shown that patient satisfaction was significantly better in rooms with cyclic lighting environments [60]. However, they have not been shown to decrease the cumulative incidence of delirium, nor to restore circadian rhythm in critically ill patients [43]. Some relevant conclusions derived from these studies for future trials are that dynamic lighting intervention should be included in a multi-component intervention of the ICU environment, that patients should be with minimal or no sedation, and that dynamic lighting should reach sufficient levels to activate the circadian system to impact relevant outcomes [54, 60].

Noise, defined as unwanted sound, could affect patients both psychologically and physiologically and is considered an important environmental factor of sleep disturbance in the ICU [19, 23, 26], especially when patients are recovering from illness and/or are more conscious [22, 23, 58]. Studies in ICUs indicate that over time, the average daytime and nighttime noise in hospitals has increased [27, 29], that daytime shifts are noisier than nighttime shifts, and that noise levels are significantly higher on weekdays than on weekends [28]. Patients perceive staff conversations and alarms during the night as the most disturbing [4–6, 22, 23, 58]. One strategy to counteract noise is auditory masking. This technique is often used to minimize distractions with other sounds in different environments. Auditory masking is a phenomenon in which the perception of a sound is reduced by the presence of another sound (sound masker) [45]. The mechanism of auditory masking is to add background noise to reduce changes in the sound from the baseline to the peak. This may influence the amplitude and latency of cerebral (cortical) evoked potentials, so that in the presence of added background noise (white or pink noise), a large acoustic stimulus may elicit less intense cerebral cortical activation during sleep [35, 36]. Noise in the natural world is classified into 3 categories according to frequency: white, pink, and brown, and they have significantly different properties and sound different to the human ear [33, 34]. In neonates and infants, auditory masking has been useful in promoting sleep [25, 32]. In normal individuals, the addition of mixed-frequency white noise substantially reduces sleep arousals, and although the mean basal sound level increases with the addition of white noise, sleep is more consolidated and arousals occur much less frequently [31, 46]. In the ICU, patients exposed to white noise improve and maintain qualitatively assessed sleep [45] and it is more effective in improving sleep than other noise reduction strategies, such as earplugs [25]. The use of pink noise instead of white noise has been proposed because pink noise has been shown to synchronize brain waves and induce brain activity to a specialized state at a lower level of complexity. This is the mechanism by which pink noise decreases brain wave complexity and induces a more stable sleep time, with less fragmentation and periods of wakefulness [36], compared with white noise.

Another reason for sleep disruption in ICU is patient care activities [18, 22, 23, 58]. ICU patients may experience 40 to 60 interruptions each night due to patient care activities. These activities include patient assessments, vital sign measurements, equipment adjustments, medication administration, phlebotomy, radiographs, wound care, transport, and bathing [37–39], and rationalization and reorganization of these activities during the night could contribute to improved sleep [47, 48]. The reorganization of patient care activities has been studied especially in the context of multifaceted interventions and has been associated with significant improvements in perceived sleep quality and the incidence of delirium [38]. Blocks of 4 h of rest may not be possible for all medical ICU patients but were able to minimize interventions to a significant degree. Because of resistance to interventions involving behavioral changes (e.g., reorganization of patient care activities), two conditions are important for the selection of guidelines: that they are easy to implement and that they do not decrease patient safety. Because these types of protocols conflict with ICU culture and practice at many levels, it is necessary to involve relevant stakeholders, recruit nurse champions, and obtain the support of hospital and ICU management to make the intervention successful.

### Rationale for proposed study

Sleep disturbances are common in the ICU and are associated with worse outcomes when they occur. Although several factors have been associated with sleep disturbances in ICU patients, no recommendation has yet been made on improving sleep and restoring circadian rhythm. Studies that have evaluated the effect of environmental interventions in the ICU suggest that certain environmental modifications may improve sleep in critically ill patients. However, these studies have been conducted in specialized ICU or simulated ICU settings, evaluating isolated interventions and not as part of multifaceted interventions. It is still being determined whether these proposed benefits would be replicated in a randomized controlled trial or would translate to multipurpose critical care.

This trial evaluates the impact of the use of a multifaceted intervention of environmental control in the ICU on the quantity and quality of sleep, delirium, and on post-intensive care neuropsychological impairment, in critically ill patients. Our primary objective is to assess the effects of the use of a multifaceted intervention of environmental control in the ICU on sleep quantity in terms of time in slow wave sleep-N3 and time in Rapid eye movement-REM, total time of sleep; on sleep quality and circadian timing. Our secondary objective is to document the effect of the multicomponent intervention on delirium (prevalence and duration of delirium in ICU). We hypothesize that in critically ill patients, the use of a multifaceted intervention of environmental control in the ICU, based on dynamic light therapy, auditory masking, and rationalization of ICU nocturnal patient care activities, is associated with improved quantity and quality of sleep assessed by polysomnography and other semi-quantitative methods, compared to standard care. We also investigate the effects of the use of a multifaceted intervention of environmental control in the ICU on neuropsychological impairment (depressive symptoms and cognitive impairment) at 6 months post-ICU discharge.

## Methods

### Design and study setting

This protocol is described as required by the 2013 SPIRIT guidelines to ensure consistent reporting of clinical trials. The trial is a prospective, parallel-group, randomized trial designed to evaluate the effects of the use of a multifaceted intervention of environmental control in the ICU on quantity and quality of sleep, delirium, and on post-intensive care neuropsychological impairment, in critically ill patients. A total of 56 patients will be enrolled, and 28 subjects will be randomized to each arm.

The study will be performed on adult critically ill patients with a requirement of mechanical ventilation for at least 72 hours, at the polyvalent Intensive Care Unit of the University Hospital of the Catholic University of Chile (Santiago, Chile). In particular, four of the 32 rooms will be modified during the first months of the project to install a parallel lighting system that allows the application of dynamic lighting, and an auditory masking system, which will be used in case the patient is randomized to the intervention group (see below). Trained research assistants will screen eligible individuals (those who meet inclusion criteria and do not meet any exclusion criteria). Patients who meet the criteria and who are hospitalized in a modified ICU room will be invited to participate in the study. However, if a patient meets the criteria, but is placed in another room, he/she can be transferred to any of the modified rooms if any of them is free.

Once the Informed Consent signature is obtained, patients will be randomized to receive a multifaceted intervention of environmental control in the ICU (dynamic light therapy, auditory masking, and rationalization of ICU nocturnal patient care activities) (intervention group) or standard care (control group). The randomization sequence will be generated by the Informatics Unit of the School of Medicine of the Pontificia Universidad Católica de Chile through a computer program and will be performed by randomized blocks of six, with a 1:1 allocation. The allocation concealment will be achieved through a central randomization in RedCAP and implemented by our institution´s informatic unit. The research staff will complete randomization. Due to the type of intervention under study the patient and the research team can´t be blinded to the group assignment. Nonetheless, statisticians and the researchers responsible for the analysis of the quantity of sleep (Polysomnography [PSG] and actigraphy), as well as those performing long-term outcome assessments will be blinded to the group allocation.

### Participants

Participants must meet all the inclusion criteria below:

1. Adult patients (≥18 years)
2. Patient under invasive mechanical ventilation for at least 72 hours
3. Patients without sedation or with superficial sedation level (SAS 3–4 by Sedation-Agitation Scale [61]), during most of the daytime within the 24 previous hours.

Participants who meet any of the following criteria will be excluded:

1. Patients who required mechanical ventilation in another episode of hospitalization in the 2 months before screening
2. Patients with primary neurological or neurosurgical disease
3. Presence of mental or intellectual disability before hospitalization or communication/language barriers
4. Pre-existing co-morbidity with a life expectancy not exceeding 6 months (eg, metastatic cancer)
5. Readmission to the ICU (patients can only be included if they are on their first ICU admission of the present hospitalization)
6. No fixed address for follow-up
7. Patients with moderate to severe visual or hearing impairment
8. Patients with known sleep disturbance before hospital admission
9. Early limitation of therapeutic effort

### Intervention

Enrolled subjects randomized to the multifaceted intervention of environmental control in the ICU will receive dynamic light therapy, auditory masking, and rationalization of ICU nocturnal patient care activities every day starting immediately after randomization and until ICU discharge.

The dynamic light system (DLS) will consist of a multi-channel LED Spectrum, which will automatically modify the different levels of illuminance required and at the same time will reproduce the different color temperatures in order to simulate daylight conditions during daytime. Through the software, the luminaries will automatically or manually modify the different LED channels for multispectral reproduction. The spectral output will cover the wavelength range from 420 nm to 730 nm. The system allows controlling the luminaires of each ICU box independently to operate as a circadian system or a lighting system configured for patient exploration or program-specific itineraries designed for each patient o UCI`s BOX. An intelligent control system will consist of: an intelligent minicomputer “Light-Hub”, the software control “MOTO”, a network power supply, and a WIFI Router to connect de Light HUB with de software. The LAMP luminaries will be controlled with a Phone, PC, or Tablet by the investigators. The intervention will be completely designed and performed by lighting specialist engineers of the Design School of the Catholic University of Chile.

The auditory masking system will provide a continuous background of digitally generated broadband (0.5–22.05 KHz) pink noise [33, 34]. The sound system will be placed near the head of the bed. This will be started (at 62 DB sound level) each night for 8 hours (10:00 pm– 5:00 am). In addition, heart rate, blood pressure, and saturation of O2 telemetry alarms will be set to a minimum volume inside the patient’s room. The intervention will be completely designed and performed by a sound specialist engineer from the Electrical Engineering Department and Music Institute of the Catholic University of Chile.

Starting the first day after inclusion, night-time patient care activities will be reorganized to minimize interruptions between 00:30 am and 5 am. For this purpose, the medication administration schedule will be organized, and the vital signs monitoring will be done continuously by medical devices without requiring to disturb the patient. As in our ICU temperature monitoring is performed with an axillary thermometer every hour, we will install a temperature sensor in the patient´s armpit for continuous measurement. Hygiene and comfort activities will be scheduled for the daytime. During the day medications, diagnostic imaging, laboratory draws, and care orders will be reviewed and retimed by the research staff in conjunction with the clinical staff. Emergency interventions will not be limited or altered. For patients receiving the intervention, research staff will provide one-to-one coaching to the bedside nurse. A record will be kept of the number and duration of nursing interventions during the night (from 22:00 to 8:00) until discharge from the ICU by the bedside nurses.

In this trial, the comparator or control group is standard care, this is the existing system in ICU. In terms of lighting, the lighting system control intervention currently installed in the ICU rooms provides a fixed light of 300 to 400 lux during the daytime and 0 to 30 lux during the nighttime. However, controls are not automatic; therefore, it depends on staff whether lights are switched on during daytime and off during nighttime. The lighting control system is an on / off switch.

Regarding the noise control intervention, the rooms do not have noise isolation. During the night, usually, the doors of the rooms remain closed. In our ICU, there is no protocol for reducing environmental noise. Although we have not recorded noise continuously in our ICU, we have registered isolated measurements like those reported in the literature [62, 63]. This is the mean average value of 60 dB during daytime and 50 dB during nighttime but with frequent peaks over 80 to 90 dB.

The nocturnal patient care activities intervention control: There is no specific protocol for organizing patient care activities during the night. The activities (schedules of drug administration and intravenous infusions, schedules of non-urgent examinations, and non-urgent procedures) are organized during the morning by the patient’s nurse according to their clinical criteria. Hygiene and comfort activities (bathing, linen changes, wound care, dressing changes) are carried out in standard schedules according to the rules of the ICU. Drug administration, examinations, and urgent procedures are performed when necessary.

Common procedures for both groups: In our unit, there is a physical and occupational therapy protocol for all patients requiring invasive mechanical ventilation; patients admitted to the study will be treated according to institutional protocols, regardless of the study group. Family members can visit patients every day during the afternoon; however, in our unit, there are no specific protocols to incorporate relatives in the care of patients.

In both groups, the unit’s protocol of analgesia and sedation will be used if the patient requires it. The level of sedation will be monitored as part of daily care by nurses in all patients every four hours using the Sedation-Agitation Scale. In our unit, benzodiazepines are not used except when the patient uses them chronically or to avoid drug and alcohol withdrawal syndrome. In case patients admitted to the study present delirium, they will be treated according to the institutional protocol for managing delirium, independent of the study group. In our unit, melatonin and other sleep inducers are not used routinely.

### Outcome measures

The primary outcome will be sleep quantity at day 3 of the intervention. Time in slow wave sleep-N3 assessed by PSG will provide the primary outcome for the trial. PSG will be performed on day 1 and 3 after randomization to ensure the clearance of drugs that could alter the architecture of sleep. A portable device will be used (Nox A1 System®, Reykjavík, Iceland). A recording will last 9 h, starting at 10:00 p.m. and ending at 07:00 a.m. The PSG will include electroencephalography (EEG) with electrodes placed according to the international 10–20 system, electromyography, electrooculography, electrocardiography and pulse oximetry will be recorded. PSG will report sleep efficiency, total sleep time, latency, the proportion of different stages of sleep, the presence of sleep fragmentation, the presence of micro-arousals, the presence of apneas, and the registration of eye and limb movements. Sleep recordings will be visually scored by a sleep specialist physician blinded to the group assignment using international criteria [64]. Although PSG is the gold standard for evaluating sleep, because it is a complex and expensive exam, it will only be done once on day one and day 3.

Secondary outcomes include the time in Rapid eye movement-REM, the total time of sleep, sleep quality, circadian timing, ICU delirium, neuropsychological impairment (depressive symptoms and cognitive impairment) at 6 months post ICU discharge, and quality of life at 6 months post ICU discharge.

The time in Rapid eye movement-REM will be assessed by PSG on day 1 and day 3 after randomization with a portable device will be used (Nox A1 System®, Reykjavík, Iceland).

The total time of sleep corresponded to the time in minutes that the subject sleeps during the day, and will be assessed by actigraphy. Actigraphy is a continuous measurement of a person’s movement through a device similar to a wristwatch (ACT-Trust^®^ AT0503, Sao Paulo, Brazil). It is an objective method of quantifying sleep and circadian rhythm [65–67]. All patients will have an actigraph placed on their non-dominant wrist immediately after randomization and until one week after ICU discharge. The main advantages of actigraphy compared to PSG are that it can be monitored continuously for several days, and the data registered is not intrinsically altered by medication (in contrast to EEG). Markers of sleep and circadian function derived from actigraphy include circadian timing of each 24 hours of greatest activity (within each 24 hours), sleep duration, sleep fragmentation, and regularity of circadian rhythmicity. All actigraphy data will be analyzed by a sleep physician blinded to the group assignment, using MATLAB (Matchworks, Natick, Massachusetts, USA).

Sleep quality will be assessed by questionnaires (Richards-Campbell Sleep Questionnaire [RCSQ] and Pittsburgh Sleep Quality Index [PSQI]). RCSQ [68] is a five-item visual analog scale that assesses sleep perception in critically ill patients. The scale assesses sleep depth, sleep onset latency, number of awakenings, time spent awake, and overall sleep quality. Patients will complete the questionnaire considering the perception of sleep over their previous night, from the second day of admission to discharge from the ICU.

The PSQI [69] is a self-report questionnaire that assesses sleep quality in the past month. It is composed of nineteen individual items that generate a score of seven components: subjective sleep quality, sleep latency, sleep duration, habitual sleep efficiency, sleep disturbances, sleep medication use, and daytime dysfunction. The sum of the scores of these seven components results in the overall score of the questionnaire. Patients will complete the questionnaire six months after discharge from the ICU.

Assessment of circadian rhythm will be evaluated through the oscillation of plasma concentration of melatonin and cortisol. Melatonin and cortisol plasmatic levels will be assessed on days 1 and 3 after randomization. Four samples will be taken each day. According to the manufacturer’s instructions, plasma melatonin levels will be measured with Melatonin ELISA Kit (ABCAM, USA).

The presence of delirium will be evaluated daily using the Confusion Assessment Method for the ICU (CAM-ICU) [70], starting immediately after randomization and until ICU discharge. Prevalence of delirium will be defined as the presence of delirium (positive CAM-ICU screening) on at least one day during the ICU stay. Duration of delirium will be defined as the total days during ICU stay.

Neuropsychological impairment (depressive symptoms and cognitive impairment) at 6 months post ICU discharge will be assessed by Hospital Anxiety and Depression Scale (HADS) [71] and Montreal Cognitive Assessment (MoCA©) [72]. HADS is a self-assessment scale used to detect emotional distress (anxiety and depression) in the non-psychiatric population. It is a short instrument (14 items) that has shown its reliability and validity in Chile, for diagnosis and to assess the severity of the disorder. It comprises two subscales (HAD-A: anxiety and HAD-D: depression) of seven items, each with scores from 0 to 3. The authors themselves recommend the original cut-off points: eight for possible cases and >10 for probable cases in both subscales. MoCA© is a questionnaire to assess global cognition function validated to detect cognitive impairment and dementia. The MoCA is a questionnaire that is administered in 10–15 minutes. It has an overall score of 30 points and, through 10 subtests, can assess attention (0–6 points), abstraction (0–2 points), visuospatial/executive skills (0–5 points), naming (0–3 points), language (0–3 points), delayed recall (0–5 points), and temporal and spatial orientation (0–6 points). The questionnaire defines cognitive impairment as a MoCA score < 26. The overall MoCA score should be adjusted in case of patients with < 12 years of education, in these cases 1 point is added to the overall MoCA score.

Outcomes assessment will be conducted by research assistants blinded to randomization assignment at baseline and six months post ICU discharge.

### Participant timeline and recruitment

**Fig 1:**
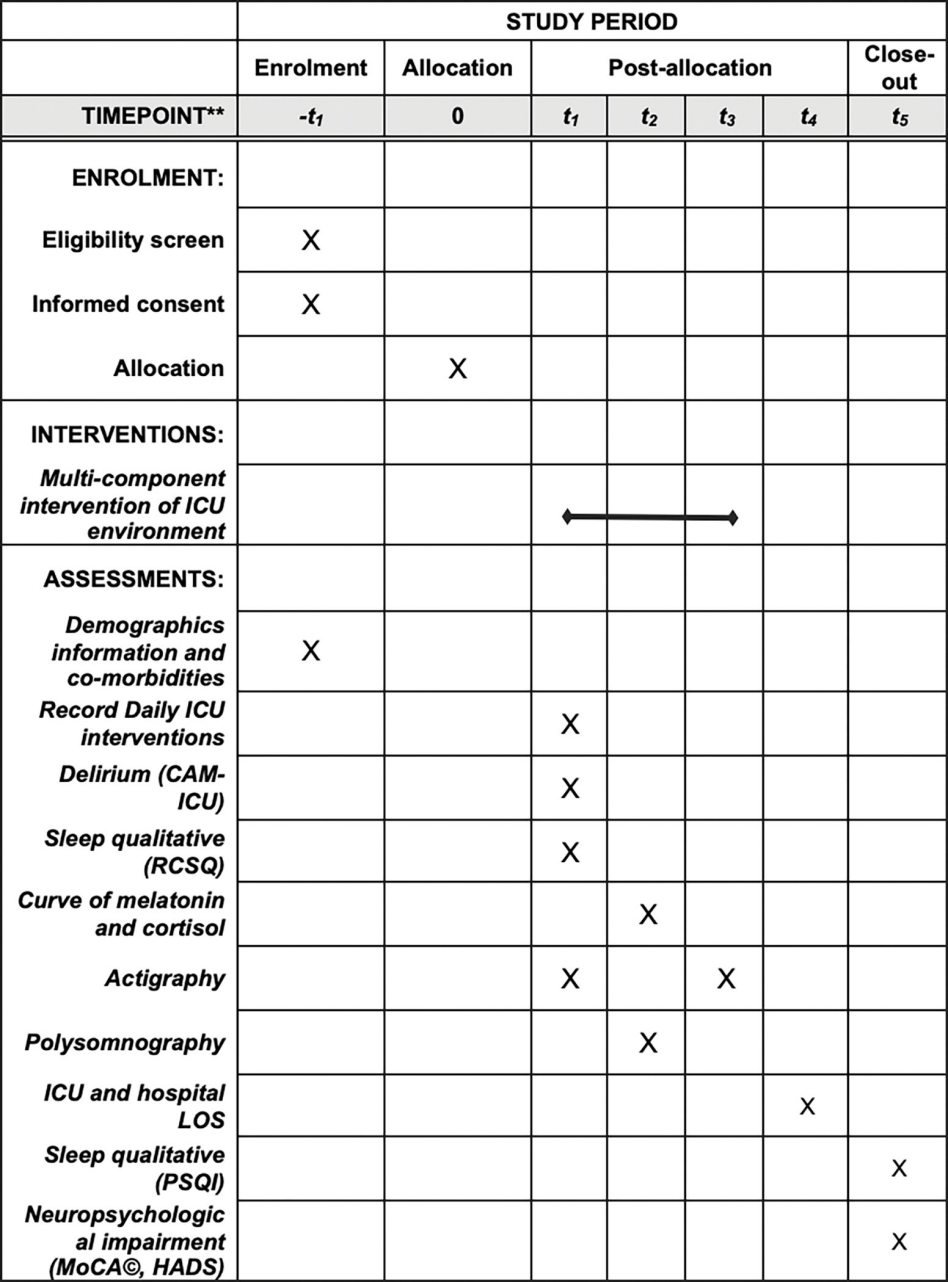
Timeline of recruitment, allocation, and assessment. *-t*_*1*_: Baseline; *t*_*1*_: Each day until ICU discharge or on day 28; *t*_*2*_: Day 3; *t*_*3*_: Day seven after ICU discharge; *t*_*4*_: Hospital discharge; *t*_*5*_: 6 months follow-up. Abbreviations: ICU *Intensive Care Unit*; CAM-ICU *Confusion Assessment Methods for the ICU*; RCSQ *Richards-Campbell Sleep Questionnaire*; PSG *Polysomnography*; LOS *Length of stay*; PSQI *Pittsburgh Sleep Quality Index*; HADS *Hospital Anxiety and Depression Scale*; MoCA *Montreal Cognitive Assessment*.

Patients will be examined in the morning to determine eligibility. Baseline data will be recorded on day 0. Patients will be assessed each day, and primary and secondary outcomes will be recorded daily until ICU discharge or on day 28 after enrollment. Patients will be assessed on the ward until day seven after ICU discharge to complete the sleep questionnaire and actigraphy. Discharged patients will be contacted by telephone. Patients will be followed up during their hospital stay to record ICU stay, hospital stay, and mortality. Patients will be evaluated at six months from ICU discharge to record secondary outcomes. The trial evaluation schedule is shown in Fig 1.

### Data collection and management

Data will be collected and stored using Research Electronic Data Capture (RED-Cap). At the time of enrolment, study staff will collect information on each patient including patient demographics, dates of hospital and ICU admission, and illness severity according to the Acute Physiology and Chronic Health disease Classification System II (APACHE II), Chronic Health Evaluation II and Sequential Organ Failure Assessment Score (SOFA), co-morbidities according to the Charlson Co-morbidity Index, admission diagnoses, and pre-existing neuropsychological impairment.

During each patient’s stay in the ICU, data will be collected on: Sequential Organ Failure Assessment Score (SOFA), Partial pressure of oxygen (PaO2)/Fraction of inspired oxygen (FiO2), daily arterial pressure and the minimum glycemic value obtained, daily doses of medications. Use of hemodialysis and vasopressors, mechanical ventilation duration, and ICU/Hospital length of stay data will be collected also. All patients will undergo a standardized follow-up immediately prior to hospital discharge and at 6 months later. At 6 months patients will attend a follow-up visit, and if they are unable to attend they will be visited at home.

A sonometer (Cirrus CR:172B, Cirrus Research, UK) and a luxometer will be placed near the patient’s head. Ambient sound will be measured continuously and illuminance will be measured every 30 minutes until ICU discharge. These data will be registered and analyzed later by light and sound experts.

The research assistant will undergo training and certification procedures with a supervisor for quality assurance. The supervisor performs ongoing quality assurance checks at regular intervals. Subjects will be instructed to refrain from discussing their assigned intervention with the research assistant.

### Statistical analyses

Multilevel models with multivariate random intercept will be used for the proposed results in the successive tests verified over time, which will be defined as linear if the result is quantitative and logistic if the result is dichotomous. In addition, the model will be adjusted for confounding factors (age, sex, socioeconomic status, comorbidities, severity score, drug use, type of feeding, days of invasive mechanical ventilation at baseline). Interaction between treatment and time will also occur, assuming that results may vary. The proposed models will be executed using the ’lme4’ library in the free software R. It is expected that there are missing values due to the deaths and withdrawals of the participants. To improve precision and reduce bias in the estimates, methods such as multiple imputations will be used to treat missing values. A sensitivity analysis will be performed, assuming different patterns of loss in the data.

According to a previous study, which measured sleep with PSG [73], and other studies, which have described sleep architecture in the ICU [3–7], we estimate that the N3 stage time in our study population will be 7 minutes with an SD of 9 minutes. A 50% improvement in N3 stage time would be both clinically relevant and realistic. With a power of 80% and with an alpha of 5%, we calculated that a sample size of 56 patients is required to demonstrate statistically significant differences.

### Monitoring

Principal investigators carry out data monitoring: they ensure the progress of research protocol, randomization, data collection, and visit organization. A research assistant helps principal investigators in the screening of patients and assessment of clinical data and outcomes. An expert statistician from the Catholic University of Chile carries out data analysis and statistics. Principal investigators meet regularly to ensure and control trial progress and are in constant contact.

Due to the intervention nature, serious adverse events are not expected. Any adverse event that occurred will be reported in the manuscript describing trial results if they are directly related to the intervention. Those adverse events will be monitored by principal investigators and addressed until resolution. The research assistant will be trained in psychological first aid and alert to signs of anxiety, emotional suffering, and frustration displayed by subjects during the outcomes assessment. They will monitor reactions closely and respond appropriately with encouragement and suggestions.

### Ethics and dissemination

The study was approved by the Institutional Review Board of the Faculty of Medicine of the Catholic University of Chile (#190528002). The principal investigator and research staff will be responsible for conducting the informed consent process with all study participants. Signed written informed consent will be obtained from the responsible family member or legally authorized representative, as appropriate, and will be subsequently confirmed by the patients when feasible. The Intensive Care Unit Director will attest to the informed consent process for each participant. Changes to the study protocol and/or the informed consent will be sent to the Institutional Review Board as protocol amendments. To protect confidentiality before, during, and after the trial, all data will be deidentified prior to analysis; only the principal investigator and project manager will be able to access the code to identify the individual. Before starting the trial, the protocol was registered on ClinicalTrials.gov NCT05694052 on January 10, 2023, and the trial status will be updated accordingly.

## Discussion

Sleep and circadian rhythm are altered in critically ill patients. Some studies have been performed testing different pharmacological and non-pharmacological strategies to improve sleep in these patients, but their results have not been consistent. Despite the emerging evidence showing multiple physiological and psychological consequences of sleep and circadian rhythm disturbances in the ICU, and some of them can significantly impact patient-centered clinical outcomes, there is still no recommendation on the best strategy to improve ICU sleep and re-establish circadian rhythm. Therefore, effective interventions to improve ICU sleep should be investigated. SLEEP-ICU represents a step forward in the study of non-pharmacological interventions in the ICU to improve sleep in critically ill patients and their long-term cognitive and psychological outcomes. It is a randomized controlled trial that examines the effect of an innovative and uniform multicomponent ICU environmental control intervention on sleep in critically ill patients and long-term cognitive and psychological well-being. Additionally, it is an easy-to-implement and low-cost intervention.

There are, however, limitations related to the sample. The data will be generated from a single ICU in Chile, limiting the findings’ generalizability. Due to its longitudinal nature, there is a risk of loss to follow-up and missing data, which would call into question the internal validity of the results reported by SLEEP-ICU. However, we have established protocols to minimize the loss of follow-up. On the other hand, blinding patients or investigators is not possible due to the nature of the intervention.

SLEEP-ICU will be the first randomized clinical trial in critically ill patients to evaluate the effect of a multicomponent, non-pharmacological environmental control intervention (lighting, noise, and patient care activities) on sleep improvement. Second, we propose an interdisciplinary and pragmatic approach that targets different environmental factors and integrates experts from various fields. Although the concepts of dynamic light and sound masking are well known, our proposal includes the local design of solutions based on these concepts. Third, this intervention is based on a plausible pathophysiologic pathway, and the collection of clinical data, biomarkers, and qualitative and quantitative assessment of sleep will help to establish the mechanism between the environment ICU, delirium, sleep, and circadian rhythm, and long term cognitive and psychological impairment in ICU survivors. Finally, we chose to use the highest standard for sleep measurements (PSG), which has been one of the significant limitations of previous research in this field. Additionally, we will examine the short-term and long-term effects of SLEEP-ICU intervention on psychological symptoms and cognition following ICU discharge and 6 months after hospital discharge, using a comprehensive battery of psychological and cognition assessments.

In summary, SLEEP-ICU is the first randomized clinical trial to evaluate the combined effect of a multicomponent environmental management strategy on quantitative and qualitative sleep improvement in critically ill adults. The results of the study will provide guidance on environmental interventions in the ICU to prevent sleep and circadian rhythm disturbances and their associated complications and will enable evidence-based selection of non-pharmacological interventions in critically ill adults in the ICU.

## Supporting information

S1 Checklist SPIRIT 2013 checklist: Recommended items to address in a clinical trial protocol and related documents. (PDF)

S1 Appendix Approval by ethics committee. Original document of approval of the clinical trial by Ethics Committee (in Spanish).
(PDF)

S2 Appendix Original protocol sent to ethics committee. Original protocol sent to Ethics Committee (in English).
(PDF)

S3 Appendix Letter of external funding. (PNG)
