# Supplementary material for: Improve sleep in critically ill patients: Study protocol for a randomized controlled trial for a multi-component intervention of environment control in the ICU
Source: PLoS One. 2023 May 25;18(5):e0286180. doi: 10.1371/journal.pone.0286180 (PMC10212109; doi:10.1371/journal.pone.0286180)
Supplement: S1 Appendix — Original document of approval of the clinical trial by Ethics Committee (in Spanish). (PDF) [file pone.0286180.s002.pdf]

PONTIFICIA UNIVERSIDAD CATÓLICA DE CHILE

ACTA DE APROBACIÓN
COMITÉ ÉTICO CIENTÍFICO CIENCIAS DE LA SALUD UC
Re-acreditado por SEREMI de Salud
Resolución Exenta N°012321 del 07 de junio de 2017

NUEVO ESTUDIO
Fecha y N° de Sesión: 11de junio de 2020, Sesión N°8
Investigador Responsable: Leyla Alegria Vargas
ID Protocolo: 190528002
Título del Proyecto: Impact of the use a multifaceted intervention of environment control in the ICU to optimize quantity and quality of sleep in critically ill patients.
Facultad/Unidad Académica: Facultad de Medicina, Pontificia Universidad Católica de Chile
Académico Responsable: Alejandro Rodrigo Bruhn Cruz
Sitio de realización: Unidad de Paciente Crítico, Hospital Clínico Red de Salud UC Christus
Financiamiento: Regular Fondecyt

Miembros del Comité que participaron en la aprobación del estudio:
Dra. Claudia Uribe Torres, Presidente.
Dra. Colomba Cofré Dougnac, Vice-Presidente.
Sr. Jorge Muñoz Castillo, Abogado miembro externo.
Dr. Gustavo Kaltwasser González, Miembro externo.
Srta. Alyssa Garay Navea, Representante de la comunidad.
EU Angelina Dois Castellón, Escuela de Enfermería.
Klgo. Antonio López Fuenzalida, Carrera de Kinesiología.
Prof. Ivonne Vargas Celis, Centro de Bioética.
Dra. Marisa Torres Hidalgo, Departamento de Salud Pública.
EU Mónica Cifuentes Soro, Miembro externo.
EU Víctor Contreras Ibacache, Departamento de Anestesiología.

Documentos recibidos por el Comité:
- Carta Presentación Investigador Responsable
- Carta Apoyo Jefe de Departamento

Documentos revisados y aprobados por el Comité:
- Formulario de Solicitud de Revisión Ética
- Documento de Consentimiento Informado
- Proyecto Fondecyt Regular

Considerando que:

1- Los investigadores referidos cuentan con la experiencia necesaria para la conducción y el desarrollo de este tipo de estudio;
2- La metodología descrita es apropiada para el cumplimiento de los objetivos del estudio, de acuerdo con los estándares internacionales de rigor científico;
3- Durante la conducción del estudio se garantiza un balance riesgo/beneficio favorable para los participantes, por cuanto sólo se realizan procedimientos de bajo riesgo;
4- Pese a que la población por estudiar se ha considerado vulnerable, por cuanto se refiere a pacientes graves en ventilación mecánica, el protocolo ha resguardado la seguridad y bienestar de los participantes;
5- Se ha contemplado el resguardo de la confidencialidad de la información sensible e identificable en la difusión de los resultados, por lo que no introduce un riesgo de menoscabo para la intimidad de los participantes; y
6- Los representantes consentirán voluntariamente luego de ser adecuadamente informados sobre los aspectos esenciales del estudio, sus deberes y derechos, y los plazos estipulados para el cumplimiento de los objetivos de la investigación.

Constatado que, el texto del documento de Consentimiento Informado contiene:

1- La descripción general de los objetivos de la investigación;
2- El detalle de los procedimientos que involucra la participación en este estudio;
3- Los antecedentes sobre el uso que se dará a la información obtenida a partir de cada procedimiento de la investigación;
4- El compromiso respecto a la utilización actual y futura de la información, la que sólo se realizará dentro de los marcos del presente estudio y para el logro de dichos objetivos;
5- El resguardo de la confidencialidad y el anonimato de la información recogida, según corresponde a cada procedimiento del estudio;
6- El detalle respecto del costo en tiempo que significa la participación en el estudio;
7- La información sobre los beneficios, derechos frente a riesgos y cobertura de daños por la participación en la investigación; y
8- La voluntariedad de la participación y la garantía para cada participante de hacer abandono del estudio, sin repercusión alguna.
9- Un párrafo de consentimiento final, para cuando el participante tenga la capacidad para consentir por sí mismo

PONTIFICIA
UNIVERSIDAD
CATÓLICA
DE CHILE

Resolución CEC-Salud UC:

Este proyecto cuenta con la opinión favorable del Comité con fecha **11 de junio de 2020**, en la **sesión ordinaria N°8**, la que tiene vigencia de un año. **Sin embargo, debido a la contingencia actual de pandemia, y en virtud del resguardo del balance riesgo beneficio favorable a los participantes, el CEC Salud UC solicita al Investigador Responsable iniciar el enrolamiento del estudio y el proceso de Consentimiento Informado presencial, una vez que las condiciones sanitarias lo permitan.**

Para iniciar el proceso de consentimiento y de reclutamiento se debe disponer previamente de la última versión aprobada y timbrada del documento de Consentimiento Informado. De este modo, el Investigador Responsable velará por la realización de estos procedimientos, utilizando las copias de cada versión original (timbradas, fechadas y firmadas por el CEC-Salud UC y/o por los CEC correspondientes).

El Investigador Responsable deberá solicitar la renovación anual de la presente aprobación ética con al menos 45 días de anticipación si desea continuar con el estudio. Si no ha recibido la respuesta oficial a su solicitud, el investigador deberá detener las actividades del proyecto, no podrá enrolar a ningún nuevo participante y no podrá proceder con el análisis de los datos.

En la eventualidad de requerir cualquier modificación al estudio o a los documentos aprobados originalmente, el investigador deberá notificarlo al Comité por medio de una enmienda (a través de plataforma) para la evaluación y emisión de una nueva acta de resolución ética.

El Investigador Responsable debe descargar, revisar, firmar y subir a plataforma, documento de Responsabilidad del Investigador, la que se encuentra disponible en sección “Plantillas”, del estudio de la referencia **(ID: 190528002)**

Comité Ético Científico UC de Ciencias de la Salud

DRA. COLOMBA COFRE DOUGNAC
Secretaria Ejecutiva(s) CEC-Salud UC

DRA. CLAUDIA URIBE TORRES
Presidente CEC-Salud UC

EN CASO DE CUALQUIER DUDA SE LE SOLICITA CONTACTARSE CON EL CEC-Salud UC

Se certifica que la información contenida en el presente documento es correcta y que refleja el Acta del Comité Ético Científico de Ciencias de la Salud UC (CEC-Salud UC). Este Comité adhiere a los principios éticos de la Pontificia Universidad Católica de Chile, que considera como eje fundamental el respeto a la dignidad de la persona humana en cualquier condición. Este Comité cumple además con las Guías de buena práctica clínica definidas por la Conferencia Internacional de Armonización (GCP-ICH); y con las leyes chilenas 19.628; 20.120; 20.584 y 20.850 que modifica el Código Sanitario
