# Supplementary material for: Improve sleep in critically ill patients: Study protocol for a randomized controlled trial for a multi-component intervention of environment control in the ICU
Source: PLoS One. 2023 May 25;18(5):e0286180. doi: 10.1371/journal.pone.0286180 (PMC10212109; doi:10.1371/journal.pone.0286180)
Supplement: S2 Appendix — Original protocol sent to Ethics Committee (in English). (PDF) [file pone.0286180.s003.pdf]

PROPOSED RESEARCH

I. Theoretical foundations and state of the art

1. Normal sleep

Sleep can be defined as a periodic and reversible state of disengagement from the environment. It consists of an active process that involves multiple, complex physiological and behavioral mechanisms of the central nervous system. Sleep is a natural process highly conserved during evolution, which is critical for health and wellbeing, being essential for rest, for repair, and for the survival of the individual. It is controlled by a circadian system that drives 24-h periodicity, and a homeostatic system that ensures that adequate amounts of sleep are obtained. Sleep is regulated by several centers in the brainstem, hypothalamus, thalamus and forebrain (1). External factors such as light/dark cycle can affect the circadian rhythm, interact with internal clocks by synchronizing their different phases, and as a consequence can affect normal sleep (1-2). High-quality, efficient sleep of adequate duration helps to consolidate memory, regulate the immune system, and coordinate neuroendocrine function. By contrast, abnormalities in sleep are thought to increase the risk of a broad range of adverse health effects, including cardiovascular disease, depression, cognitive impairment, seizures, and even overall mortality (1-4).

Sleep can be assessed in terms of quantity (total sleep time and time spent in each sleep stage), quality (fragmentation, sleep stage changes, wake after sleep onset, EEG sleep patterns), and distribution over the 24-h cycle. Polysomnogram (PSG) is often considered the standard criterion for sleep measurement (1,2,9). PSG defines and divides the sleep into nonrapid eye movement (NREM) sleep and rapid eye movement sleep (REM). According to the newly released scoring criteria, NREM sleep is now characterized by three substages: N1, N2 and N3 (N3 substage is also known as slow wave sleep [SWS]) (1,9). Substages N1 and N2 correspond to light sleep, while N3 corresponds to deep sleep. N3 sleep is associated to respiratory and cardiovascular stability, and is considered the most important stage of sleep for physiological repair, for bone and muscle build up, and for immune system recovery. The importance of REM lies in that it is a period of memory consolidation during which important memories are conserved and non-relevant neuronal connections are eliminated (9). Alternative surrogate techniques have been utilized to assess sleep such as actigraphy, which measures the level of activity at the wrist to discern wakefulness, judged by movement, from sleep, estimated by stillness. In the same way, circadian rhythms can be assessed through chronobiologic analysis of the time series of melatonin, cortisol and temperature (1,2).

2. Sleep in critical illness

Sleep and circadian rhythms are markedly disturbed in intensive care unit (ICU) patients. These abnormalities include sleep deprivation and disruption as well as abnormal architecture, and abnormal oscillations of melatonin, cortisol, and body temperature (3-8). Sleep disruption is one of the most frequent complaints from ICU patients (5,8). In terms of sleep architecture, severe sleep-wake disorganization and alterations in sleep stage distribution are characteristic in ICU patients. Studies using PSG have demonstrated prolonged sleep latency, sleep fragmentation, decreased sleep efficiency, numerous arousals, a preponderance of substage N2 sleep, decreased or absent N3 (deep) sleep, and decreased or absent REM sleep (3-8). Although mean total sleep time does not differ markedly from healthy adults, approximately 50% of ICU sleep occurs during daytime hours, with a marked shift toward light stages of sleep (3,6,8).

In the ICU, PSG is also considered the criterion standard criterion for sleep measurement (3,7,8). Alternative surrogate techniques in the ICU include actigraphy and Bispectral index (BIS), an electroencephalogram-derived method for assessing the depth of sedation, mainly used in the operating room. Unfortunately, BIS monitoring has still not yet shown clinical benefits in ICU. Likewise, subjective survey instruments, such as the Richards-Campbell Sleep Questionnaire (RCSQ) and Pittsburgh Sleep Quality Index (PSQI), have been investigated for ICU sleep measurement, however, there remains concerns with patient´s self-reports while sedated or delirious (7-8).

Sleep disruption in critical patients is associated with several adverse consequences increasing the risk of adverse hospital outcomes, including mortality (12,13)(16-18). Other adverse hospital consequences involve poor respiratory function, impact on the cardiovascular system, aberrant immune system activation, and profound alterations on metabolism and endocrine function (6,13,16,18). Disrupted sleep can also result in added anxiety and pain in ICU patients. Evidence shows that the loss of 4 hours of sleep, and particularly the loss of REM-specific sleep, is associated with hyperalgesia symptoms the following day (13,15). During prolonged sleep deprivation, there is an increase in self-reported feelings of depressed mood, anger, frustration, tension, and anxiety (14,15). Also, sleep deprivation significantly affects cognitive function. An association between delirium and sleep disturbance has been suggested, as

prolonged sleep deprivation over several days can trigger perceptual distortions and hallucinations in healthy individuals, and all these effects may play a role in the frequent occurrence of delirium in ICU patients (3-9)(16,17). Both conditions share a number of important mechanisms, risk factors, and symptoms, however, this relationship remains debated (3-8).

Moreover, sleep disturbances are not restricted to the ICU stay but may remain even after discharge from hospital in ICU survivors. The prevalence of sleep disturbance in post-ICU patients is high, ranging from 22 to 57% at 3 months after hospital discharge (10,14,18). Studies at 6 months after ICU discharge show that patients continue to experience little or no SWS and/or REM sleep, independent of external factors (3,7,13), which may impair recovery (10,14). In the same way, persistent sleep disturbances post hospital discharge have been consistently associated with psychological comorbidities such as depression (18-21), but the underlying mechanism between sleep, circadian rhythm disruption, and depression, is not well understood. Finally, chronically reduced sleep also leads to substantial decrements in health-related quality of life, suggesting that ICU-related sleep disruption may contribute to the low quality of life observed in ICU survivors (20-21).

Major factors for sleep deprivation in critically ill patients are the type and severity of underlying illness, the pathophysiology of the acute illness, pain (from procedures or the underlying condition), and stress/anxiety (6). Mechanical ventilation (MV) may also contribute to sleep disruption in critically ill patients. Sleep in mechanically ventilated patients is highly fragmented (6-12). However, studies that have examined PSG in these patients have observed the same patterns of sleep architecture as in patients without MV (9-12). Similarly, several commonly used ICU medications, including vasopressors, antibiotics, sedatives, opioids, and analgesics, may have profound effects on sleep quantity and quality (6,9-12). Although these factors related to patients' condition play a large role in sleep disruption, they are difficult to modify. However, factors associated to the ICU environment have also been reported to have a major role in sleep disturbance (e.g, lighting, noise, patient care activities), but they can be modified by changes in ICU design and culture (20).

3. Impact of the ICU environment on sleep

The ICU can be a hostile and stressful environment for patients. From the point of view of ICU survivors, a stay in the ICU represents a traumatic event in their lifes . The ICU environment is disruptive and does not favor rest. It is a busy environment, ICU-staff works 24-h continuously with a high workflow. ICU also is a noisy environment due to the equipment (alarms and sound from monitors and life-supporting devices) and human factors (staff conversations), related to therapy and care (5,19,20)). A night in a typical ICU has been described as a "chorus of alarms, voices, and telephones rings, direct and indirect light pollution, and interruptions from unfamiliar care providers" (22). These aspects can be intensified during emergency situations, which can occur both during the day and at night. In this context, several environmental factors that could contribute to alter patients' sleep can be identified. Surveys carried out on patients and quantitative measures indicate that the main environmental factors that produce alterations in sleep are light, noise and interruptions due to care activities (23-26).

Effects of light on abnormal sleep in the ICU

The light-dark cycle is probably the most powerful entraining factor in the human circadian rhythm and the sleep-wake cycle (1,2). The ability of light to modulate cortical activity and circadian rhythm is defined, in part, by the duration, intensity (lux [lx]) and wavelength (nm) of the lighted stimulus (29,34). Artificial light can also modulate cortical activity according to color temperature (K, kelvin degrees), corresponding to the color of the light emitted by a luminaire. Warmer color temperatures (ranging from < 2700K to 3500K) represent daylight hours when the sun is rising or setting, and when people should be waking up or falling asleep (29). Endogenous melatonin secretion occurs in parallel with the light-dark cycle, with melatonin concentrations reaching low values during daytime and high values at night (28,34). Therefore, indoor lighting system should be dynamic, providing greater intensity and color temperatures during the morning, to support the awakening, decreasing to a more standard intensity later down, and finally in the afternoon, reduce the intensity of light to promote melatonin secretion (29,34). In ICU, the primary purpose of lighting has been to support the treatment and monitoring of patients with severe disease. However, it has been proposed that lighting could be used to favor a home atmosphere that allows calming the patient, and facility recovery. However, light is also a major cause of sleep disruption in the ICU setting (37). Bright light (≈ 7,000 to 13,000 lux) is well recognized as an effective circadian synchronizer, however the pacemaker is also sensitive to low intensity light (≈ 180 lux) (36). The European lighting standards (DIN EN 12464-1) recommend lighting levels of at least 300 lux for simple examinations and 1,000 lux for bedside treatments and emergency in the ICU, and at night the illuminance should not exceed 20 lux (29,36). Studies that have measured light in the ICU have generally documented levels

between 300 and 500 lux during the day, and between 0 and 145 lux in the night (20). A light level of ≈ 1,000 lux is required to suppress melatonin to nearly daytime levels (28,33,34). Nocturnal exposure to light levels between 100 and 500 lux decreases the secretion of melatonin, which can ultimately result in sleep disruption (3,9,27), and have an important effect on the circadian pacemaker (34).

Effects of environmental noise on abnormal sleep in the ICU

Noise, defined as unwanted sound, could affect patients both psychologically and physiologically. Noise is considered a major environmental factor for sleep disruption in the ICU (20,25). When the ICU patients are recovering from illness and/or they are more cognitive, noise may be perceived as a more substantially disruptive factor (24,25,27).The distribution of frequencies in the sound spectrum, a mathematical relationship between frequency and sound level, is important for sound perception. The Environmental Protection Agency recommends hospital noise levels should be below 40 dB (42,44). However, one study indicated that in the half century since 1960, average daytime noise in hospitals has increased from 57 dB in 1960 to 72 dB by 2005, whereas average nighttime noise has increased from 42 dB in 1960 to 60 dB by 2005 (46). Other study performed in ICU concluded that day shifts are noisier than the night shifts and the sound levels were significantly higher during weekdays than on weekends (45). Summarizing, the noise levels in ICUs have been found to be elevated with average daytime sound peaks of 150 to 200 dB and nighttime peak > 80 dB (42-46). PSG studies in ICU patients show that only 10 to 30% of arousals can be attributed to noise. Nonetheless, according to the literature, among specific sources of noise, staff talking accounted for 26% of the noise, had the highest peak decibel level, and was perceived to be the most disruptive (4-6). Alarms are also reported as one of the the most disturbing noises in ICU (24,25,27). It is interesting to note that alarms are not usually perceived as helpful by the ICU staff (43).

Effect of staff-patient interactions on abnormal sleep

Other reason for sleep disruption is patient care (11,24,25,27). In an observational study, fifty records of patients from four ICUs were revised in order to establish the number of times nurses performed procedures with patients during the night, over 147 nights. They found that there were more interactions with patients at midnight and less at around 03:00 hrs. in the morning. Interestingly, only 9 out of 147 nights had a 2-3 hour period of uninterrupted sleep (54). ICU patients may experience 40 to 60 interruptions each night due to patient care activities. These activities include patient assessments, vital sign measurements, equipment adjustments, medication administration, phlebotomy, radiographs, wound care, transportation, and bathing (54-56). Patients refer that vital signs control and phlebotomy are even more disruptive to sleep than noise and light (24,25,27).

Descriptive studies have defined and expanded our understanding about the relation between environmental problems and serious sleep disturbances in ICU (42-57). Consequently, a number of studies have evaluated interventions targeting sleep optimization in the ICU, including non-pharmacologic and pharmacologic interventions (22,31,32,35,38,40,41,47,58,59). Medications specifically used for acute sleep disturbances should be used for short periods with ongoing reassessment of necessity due to adverse effects (3-8). Non-pharmacologic interventions, and particularly those directed to improve the ICU environment, should be the main focus of future efforts.

4. ICU environment intervention as strategy for sleep protection in the ICU

A limited number of studies have evaluated interventions targeting sleep optimization in the ICU, including non-pharmacologic sleep bundles, bright light therapy, earplugs, eye masks, relaxation techniques and differing modes of MV, with mixed results (22,31,32,35,38,40,41,47,58,59).

The most studied strategy to date has been to isolate the patients from their environment by using eye masks and / or earplugs. These devices are perceived as easy to use, economic, and their use can be incorporated routinely into nursing activities (31,32). Several studies have been published and even systematic reviews have been carried out. Despite the significant variations of the methods, most of these studies suggest that the use of the intervention has a positive effect on sleep quality, evaluated subjectively by questionnaires. Among the main limitations of these studies are that the number of monitoring nights varies among them (1 to 8 nights), with positive effects of the intervention only in the first days of ICU (31,32,60,62). In addition, most of them have been performed in specialized ICUs with lower severity of disease (such as coronary, gynecological, or post-operative ICUs), or in simulated ICU settings with healthy individuals. Among the studies in critically ill patients, few of these studies have made objective evaluations of sleep (e.g. PSG), using mainly questionnaires which give subjective information (60,62). In the studies performed in healthy individuals exposed to a simulated ICU the use of earplugs and face masks increased REM sleep and deceased REM latency (61). However, the extrapolation of this

experimental setting to critically ill patients is extremely debatable. Another relevant issue is patient comfort with the intervention. Several studies report poor tolerance to both eye masks and earplugs, with a significant proportion of patients removing the devices due to anxiety related to isolation, pain related to the earplugs, and sense of claustrophobia with the eye masks (31,32,60,62). One of the most relevant studies in terms of quality was a single-center randomized controlled trial that included 64 mixed ICU patients, and which evaluated the impact of eye masks and earplugs on sleep architecture, measured by PSG (63). Although they observed that long awakenings (> 1 min) decreased, the primary outcome (proportion of N3 sleep) was not modified. A major limitation of this study is that only 21 out of 32 patients in the intervention group wore the earplugs and eye mask all night long, while the other 11 did not tolerate them. In a post hoc analysis the subgroup of patients who did tolerate the earplugs and eye masks exhibited improvements in most sleep parameters.

A second strategy directed to light has been to apply dynamic light in ICU rooms. As light is such an important factor in the regulation of sleep and wakefulness, it makes sense to try to restore temporal disorganization of circadian pacemakers by modulating light exposure in the ICU. In fact, light has been applied therapeutically as a treatment for sleep disorders in other patient populations (21,22). Although no study has assessed directly the effect of an improved lighting system on sleep in ICU, a couple of studies have tested the impact of dynamic light on other related outcomes. A pilot study evaluated patients´ experiences and circadian rhythm with a cycled lighting environment (38). The study could not show a clear impact on the circadian rhythm, but the patients' satisfaction was significantly better in rooms with cycled lighting environment. The most relevant study published to date was a large randomized, controlled, single-center trial performed in ICU patients at an early phase of their critical illness (n=734) (35). Patients were randomized to high-intensity dynamic light application (DLA) or usual care, and the primary outcome was the development of delirium, a frequent and relevant complication in critically ill patients. Although the intervention significantly modified light intensities, as planned, there was no difference in the cumulative incidence of delirium. The authors also evaluated melatonin metabolite 6-sulfatoxy-melatonin and the stress hormone cortisol in urine, as markers of circadian rhythm, but they observed no differences between DLA and control group, neither during the night-time, nor during morning periods. The excretion of melatonin and cortisol in both groups was abnormal compared with healthy individuals and similar to those found in other studies. Unfortunately, sleep was not evaluated in this study, although the theoretical background was that one of the main mechanisms through which DLA would prevent delirium was through sleep optimization. This negative result could be explained first, because most patients were sedated and had their eyes closed during the acute disease phase, and closed eyes could have prevented some biological effects of lighting. Second, sedation and acute illness can disturb the normal circadian rhythm, which might have precluded the effects of lighting therapy (39). Third, the lighting system used in the study might not have been biologically effective because the peak illuminance achieved during the day may have been insufficient regarding melatonin suppression (37). Some relevant conclusions derived from this study for future trials are that light interventions should be included in a multifaceted intervention, that patients should be on minimal or no sedation, and that dynamic light must reach sufficient levels to trigger the circadian system in order to impact relevant outcomes (39).

A third strategy which has been tested to optimize sleep in ICU is to decrease ICU noise during the night. A quasi-experimental study (before and after) was performed in 42 ICU patients. The intervention included: to close room doors, to decrease the volume of the telephone rings, to check and lower the volume of bedside monitor alarms, to respond to alarms within one minute, to lower the volume of staff conversation after 11 p.m. Results showed a significant decrease in nocturnal noise in the ICU and significant improvement in sleep quality assessed by questionnaires (59). Interestingly, in a survey to 1,223 healthcare providers they declare that the control of environmental noise is very important to promote sleep, but only 47% of the participants take action to reduce this noise (23). These findings suggest that some modifications to improve sleep are theoretically feasible to implement, but they require a cultural change difficult to maintain a long time.

An alternative strategy to counteract noise is auditory masking. This technique is often used to minimize distractions with other sounds in different settings. Auditory masking is a phenomenon in which the perception of a sound is reduced by the presence of another sound (sound masker) (40). The mechanism of auditory masking consists in adding a background noise to reduce the changes in sound from baseline to peak. This may influence the amplitude and latency of brain (cortical) evoked potentials, such that in the presence of added background noise (white or pink noise), a large acoustic stimulus may cause less intense cerebral cortical activation during sleep (52,53). Noise in the natural world is classified in 3 categories according to frequency: white, pink and brown, and they have significantly different properties and sound different to human ears (50,51). The color of sound refers to the power spectrum of the noise signal, and it's derived from a loose analogy between the spectrum of sound wave frequencies.

White noise contains all audibles frequencies distributed uniformly throughout the spectrum, while the pink noise has equal energy per octave (that is, the same energy from 100 to 200 Hz than it does from 10,000 to 20,000 Hz), and its spectrum has equal power in bands that are proportionally wide which results in a larger representation of low frequency noise (51).

In neonates and infants, auditory masking has been useful in promoting sleep (42). Satanchina studied 4 normal individuals and exposed them to recorded ICU noise. He found that the addition of mixed frequency white noise substantially reduced arousals from sleep and returned sleep architecture to baseline values in normal individuals, and although the mean baseline level of sound was increased with the addition of white noise, sleep was more consolidated and arousals occurred much less frequently (41). Another study performed in a coronary ICU showed that patients exposed to white noise improved and maintained sleep, although sleep was only evaluated qualitatively (40). A clinical review about the effectiveness of noise reduction strategies in ICU showed that auditory masking on average improved sleep a 42.7% compared to with earplugs (25,3%) and behavioral modification (16,1%) (42). Other studies have proposed the use of pink noise instead of white, given that its frequency content is perceived a more linear fashion in terms of its amplitudes due to the spectral power density, compared with white noise, decreased by 3 dB per octave (50-53). In addition, pink noise has demonstrated that it could synchronize brain wave and induce brain activity into a specialized state, in a lower complexity level. This is the mechanism by which pink noise decreases brain wave complexity and induces more stable sleep time with less fragmentation and wake periods (53), compared with white noise.

Likewise, patient care activities are also noted among the factors responsible for sleep disruptions in the critically ill (3-7), and the rationalization and re-organization of them during the night could contribute to improve sleep (58,59). The re-organization of patient care activities has been studied mostly in the context of multifaceted interventions. In a medical ICU a multifaceted sleep-promoting strategy was studied which included among other interventions, grouping of care activities. They found that the strategy was feasible and associated with significant improvements in perceived sleep quality and delirium incidence (55). Other study (58) incorporated into the ICU daily routine a clustered-care intervention that provides a rest period between 00:00 and 04:00 (*Naptime)*, they found that 4-h blocks of rest may not be possible for all medical ICU patients, but they were able to minimize the interventions to a significant degree. Post-intervention surveys indicated that in 38% of patients there were 3-4 room entries during the *Naptime* due mostly to nonurgent laboratory orders and urgent critical care (continuous hemodialysis, titration of vasopressors, and agitated delirium). In both studies barriers related to ICU culture were identified such as the belief that the day shift team would be frustrated because certain activities (e.g. the bathing) would have to be carried out during the day. Because of the resistance to interventions that involve behavioral changes (e.g. Naptime or re-organization of patient care activities) two conditions are important for the selection of guidelines: they must be easy to implement and they must not diminish patient safety. As this type of protocols conflicts with ICU culture and practice on many levels, relevant stakeholders must be involved, nurse champions must be recruited, and support from hospital and ICU leadership must be gained, in order to achieve success of the intervention.

5. Relevance of the present study

Although several factors have been associated to sleep disturbances in ICU patients, a recommendation about the best strategy to improve sleep and re-establish the circadian rhythm has not yet been made. As sleep deprivation in ICU may contribute to relevant clinical outcomes, effective strategies to improve sleep are worth to be investigated. We propose an interdisciplinary and pragmatic approach which targets different environmental factors, and which integrates experts from different fields. Although the concepts of dynamic light and sound masking are well known, our proposal includes the local design of solutions based on these concepts. We also chose to use the highest standard for sleep measurements (PSG), which has been one of the major limitations of previous research in this field.

We expect that our intervention will be effective to improve sleep. Although this study in underpowered to study potential impacts of the strategy on mortality or ICU length of stay, if the intervention proves to be effective in improving sleep, a larger scale study could follow. But even if the intervention proves not to be effective, the data collected about sleep and about long-term neuropsychologic outcomes in our ICU population will be highly relevant to advance our understanding about the relation between these variables, and for the planning of future studies.

II. Hypothesis and objectives

Hypothesis

In critically ill patients, the use of a multifaceted intervention of environmental control in the ICU, based on dynamic light therapy, auditory masking, and rationalization of ICU nocturnal patient care activities, is

associated with improved quantity and quality of sleep assessed by polysomnography and other semi-quantitative methods, compared to standard care.

General goal
To compare the effects of the use of a multifaceted intervention of environmental control in the ICU, based in dynamic light therapy, auditory masking, and rationalization of ICU nocturnal patient care activities, versus standard care, on quantity and quality of sleep, on delirium, and on post-intensive care neuropsychological impairment, in critically ill patients.

Specific goals
1. To compare the effects of the use a multifaceted intervention of environmental control in the ICU versus standard care, in quantity and quality of sleep by PSG and actigraphy, and by questionnaires, during the whole stay in ICU or up to day 30, and at hospital discharge and 6 months after ICU discharge.
2. To compare the effects of the use a multifaceted intervention of environmental control in ICU versus standard care, in the oscillation of plasma concentrations of Melatonin and Cortisol during the ICU at days 1 and 3 after randomization.
3. To compare the acute effects of the use a multifaceted intervention of environmental control in the ICU versus standard care, on prevalence and duration of delirium during ICU stay.
4. To compare the effects of the use a multifaceted intervention of environmental control in the ICU versus standard care, in neuropsychological impairment (depressive symptoms and cognitive impairment) at 6 months after ICU discharge.

Primary outcome
1) Sleep quantity (time in slow wave sleep-N3 and time in Rapid eye movement-REM) assessed by PSG at day 3

Secondary outcomes
1) Total time of sleep assessed by actigraphy
2) Sleep quality assessed by questionnaires (RCSQ and PSQI)
2) Circadian timing: Oscillation of plasma concentration of Melatonin and Cortisol
3) ICU delirium (prevalence and duration of delirium in ICU) by Confusion Assessment Method for the ICU (CAM-ICU)
4) Neuropsychological impairment (depressive symptoms and cognitive impairment) at 6 months post ICU discharge by cognitive testing and instrument screens of depression

III. Methodology

a. Study design
Prospective, parallel-group, randomized trial in critically ill patients. Our project will follow the CONSORT (64) guidelines for this class of studies.

b. Setting
The study will be performed at the polyvalent Intensive Care Unit of University Hospital of the Pontificia Universidad Católica de Chile. In particular, four of the 32 rooms will be modified during the first months of the project to install a parallel lighting system that allows the application of dynamic lighting, and an auditory masking system, which will be used in case the patient is randomized to the intervention group (see below).
The study will have to be approved by the Institutional Review Board of the Facultad de Medicina UC. A signed informed consent will be asked to the next of kin of all eligible patients, and confirmed later by the patients when feasible.

c. Patients
Adult patients (≥18 years) will be screened for the following inclusion criteria:
<u>Inclusion criteria:</u>
- Patient under invasive mechanical ventilation for at least 72 hours
- Patient without sedation or with superficial sedation level (SAS 3-4 by Sedation-Agitation Scale [65]), during most of the daytime within the 24 previous hours

<u>Exclusion criteria:</u>

- Patient who required mechanical ventilation in another episode of hospitalization in the 2 months before screening
- Patients with primary neurological or neurosurgical disease

- Presence of mental or intellectual disability prior to hospitalization or communication/language barriers
- Pre-existing comorbidity with a life expectancy not exceeding 6 months (eg, metastatic cancer)
- Readmission to the ICU (patients can only be included if they are on their first ICU admission of the present hospitalization)
- No fixed address for follow-up
- Patients with moderate to severe visual or hearing impairment
- Patients with known sleep disturbance before hospital admission
- Early limitation of therapeutic effort

d. Randomization

Patients who meet criteria and who are hospitalized in a modified ICU room will be invited to participate in the study. However, if a patient meets criteria, but is placed in another room, he/she can be transferred to any of the modified rooms if any of them is free. Once the Informed Consent signature is obtained, patients will be randomized to receive a multifaceted intervention of environmental control in the ICU (dynamic light therapy, auditory masking, and rationalization of ICU nocturnal patient care activities) (intervention group) or standard care (control group). The randomization sequence will be generated by a computer program with an allocation 1:1. Due to the type of intervention under study the patient and the research team can´t be blinded to the group assignment. Nonetheless, statisticians and the researchers responsible for analysis of PSG and actigraphy, as well as those performing long-term outcome assessments will be blinded to the group allocation.

e. Intervention

- **Intervention group**

Lighting: A dynamic lighting system (DLS) will be implemented. The DLS, on the basis of literature (35,37,39), will automatically modify the different levels of illuminance required and at the same time reproduce the different color temperatures in order to simulate daylight conditions during daytime. The illuminance will vary between 500 to 1,000 lux when looking straight up on the ceiling and 800 to 1,500 lux when looking towards the lamp. The DLS will provide sufficient illuminance for medical inspection (≥ 1,000 lx) even without looking directly into the light source. This system will be composed by:

a) Four light emitting diode (LED) recessed luminaires of 600x600 mm (PowerBalance RC464B LED80S, Philipps), with adjustable white features from 2700 to 6400 K and different lumen levels up to 8,000 lm (annex 2), which will be placed in the center of the ceiling. A dedicated control system will induce dynamic predefined curves that vary the temperature and intensity of the color temperature according to the hours of the day.
b) A RGB color system cove perimeter in the ICU room, which provides a basic lighting for night at 20 lux requirement, and other comfort effects (annex 4).
c) An intelligent control system will be installed which consists of: multipurpose modular room controller, dry-contact interface and LED driver, network power supply, integration devices. The DLS will be controlled from a central module, which will be only accessible by the investigators. The intervention will be completely designed and performed by lighting specialist engineers of Escuela de Diseño of Pontificia Universidad Católica de Chile.

Noise: The auditory masking system will provide a continuous background digitally generated broadband (0.5-22.05 KHz) pink noise (50,51). The sound system will be placed near the head of the bed. This will be started (at 62 DB sound level) each night for 8 hours (10:00 pm – 5:00 am). In addition, heart rate, blood pressure and saturation of O2 telemetry alarms will be set to a minimum volume inside the patient's room. The intervention will be completely designed and performed by a sound specialist engineer of the Electrical Engineering Department and Music Institute of the Catholic University of Chile.

Nocturnal patient care activities: Starting the first day after inclusion, night-time patient care activities will be re-organized to minimize interruptions. For this purpose, the medication administration schedule will be organized, and the vital signs monitoring will be done continuously by medical devices without requiring to disturb the patient. As in our ICU temperature monitoring is performed with an axillary thermometer every hour, we will install a temperature sensor in the patient´s armpit for continuous measurement. Hygiene and comfort activities will be scheduled for daytime. During the day medications, diagnostic imaging, laboratory draws, and care orders will be reviewed and retimed by the research staff in conjunction with the clinical staff. Emergency interventions will not be limited or altered. For patients receiving the intervention, research staff will provide one-to-one coaching to the bedside nurse. Number and length of nurse interventions during the night (from 10:00 p.m. to 8:00 a.m.) will be recorded until ICU discharge by bedside nurses.

- **Control group**

Lighting: The standard lighting system currently installed in the ICU rooms (annex 1) provides a fix light of 300 to 400 lux during daytime, and 0 to 30 lux during night-time. However, controls are not automatic and therefore it depends on staff whether lights are switched on during daytime and switched off during nighttime. The lighting is composed of:

a) 3 linear recessed lighting luminaires each with one fluorescent tube of 28W/T5/840, color temperature 4000K, neutral white, and a diffuser of transverse lamellae for glare control protection
b) 2 pieces of recessed compact fluorescent downlights with luminaires 2x26W/840, color temperature 4000K, neutral white
c) 1 piece of fluorescent lighting luminaire (kept on for night use) surface mounted to the driver's tray, behind the clinical bed. Fluorescent lamp 14W/ T5/830, color temperature of 3000K, warm white, with opal diffuser.
d) The lighting control system is an on / off switch, separated into three commands (see annex 1).

Noise: The rooms do not have noise isolation. During the night usually the doors of the rooms remain closed. In our ICU there is no protocol for reducing environmental noise. Although we have not recorded noise continuously in our ICU we have registered isolated measurements similar to those reported in the literature (77,78). This is mean average values of 60 dB during daytime and 50 dB during nighttime, but with frequent peaks over 80 to 90 dB

Nocturnal patient care activities: There is no specific protocol for the organization of patient care activities during the night. The activities (schedules of drug administration and intravenous infusions, schedules of non-urgent examinations, and non-urgent procedures) are organized during the morning by the patient's nurse according to their own clinical criteria. Hygiene and comfort activities (bathing, linen changes, wound care, dressing changes) are carried out in standard schedules according to the rules of the ICU. Drug administration, examinations and urgent procedures are performed when necessary.

- **Common procedures for both groups**

To avoid bias, in both groups the protocol of analgesia and sedation of the unit will be used if the patient requires it. Level of sedation will be monitored as part of daily care by nurses in all patients every four hours using the Sedation-Agitation Scale. In our unit benzodiazepines are not used except when the patient uses them chronically or to avoid drug and alcohol withdrawal syndrome. In case patients admitted to the study present delirium, they will be treated according to the institutional protocol for management of delirium, independent of the study group. In our unit, melatonin and other sleep inducers are not used routinely.

f. Study variables

1. **Baseline parameters to be collected:** At the time of enrolment, information collected on each patient will include the following: patient demographics, dates of hospital and ICU admission, illness severity according to the Acute Physiology (APACHE II), Chronic Health Evaluation II and Sequential Organ Failure Assessment Score (SOFA), comorbidities according to the Charlson Comorbidity Index, admission diagnoses, and pre-existing neuropsychological impairment.

2. **Repeated assessments to be performed during the study protocol:** During the course of each patient's stay in the ICU data will be collected on: Sequential Organ Failure Assessment Score (SOFA), Partial pressure of oxygen (PaO2)/Fraction of inspired oxygen (FiO2), daily arterial pressure and minimum glycemia value obtained, daily doses of medications. Use of hemodialysis and vasopressors, mechanical ventilation duration and ICU/Hospital length of stay data will be collected also. All patients will undergo a standardized follow-up immediately prior to hospital discharge and at 6 months later. At 6 months patients will attend to a follow-up visit, and if they are unable to attend they will be visited at home.

Quantitative assessment of sleep

- Polysomnography, will be performed on day 3 after randomization to ensure the clearance of drugs that could alter the architecture of sleep. A portable device will be used (Nox A1 System®, Reykjavík, Iceland). Recording will last 9 h, starting at 10:00 p.m. and ending at 07:00 a.m. PSG will include electroencephalography (EEG) with electrodes placed at O1/A2 and C4/A1 according to the international 10-20 system, electromyography (electrodes will be located on the levator menti muscle), electrooculography (left superior canthus, right inferior canthus), electrocardiography, and pulse oximetry will be recorded. PSG will report sleep efficiency, total sleep time, latency, proportion of different stages of sleep, presence of sleep fragmentation, presence of micro-arousals, presence of apneas, registration of eye and limb movements. Sleep recordings will be visually scored by a sleep

specialist physician blinded to the group assignment using international criteria (66). Although PSG is the gold standard for evaluating sleep, because it is a complex and expensive exam, it will only be done once on day 3.

- Actigraphy, is the continuous measurement of an individual´s movement using a wristwatch-like device (ACT-Trust® AT0503, Sao Paulo, Brazil). It is an objective method of quantifying sleep and circadian rhythm (67-69). All patients will have an actigraph placed on their non-dominant wrist immediately after randomization and until 1 week after ICU discharge. The main advantages of actigraphy compared to PSG are that it can be monitored continuously through several days, and the data registered is not intrinsically altered by medication (in contrast to EEG). Markers of sleep and circadian function derived from actigraphy include: circadian timing of each 24 hours of greatest activity (within each 24 hour period), sleep duration, sleep fragmentation, and regularity of circadian rhythmicity. All actigraphy data will be analyzed by a sleep physician blinded to the group assigment, using MATLAB (Matchworks, Natick, Massachusetts, USA).

Qualitative assessment of sleep

- Richards-Campbell Sleep Questionnaire (RCSQ)(70): this is a five-item, visual analogue scale designed to assess the perception of sleep in patients who are critically ill. The scale evaluates perceptions of depth of sleep, sleep onset latency, number of awakenings, time spent awake and overall sleep quality. Patients will complete the questionnaire as they reflect on their previously night, starting from the second day of enrollment until ICU discharge.
- Pittsburgh Sleep Quality Index (PSQI)(71): the PSQI is a self-rated questionnaire, assessing sleep quality over a 1-month time interval. Nineteen individual items generate a seven-component score: subjective sleep quality, sleep latency, sleep duration, habitual sleep efficiency, sleep disturbances, use of sleeping medications and daytime dysfunction. The sum of scores for these seven components yields one global score. Patients will completed the questionnaire at 6 months post ICU discharge.

Assessment of circadian rhythm : Melatonin and cortisol plasmatic levels will be assessed at the day 1 and 3 after randomization. Four samples will be taken each day. Plasmatic melatonin levels will be measured with Melatonin ELISA Kit, according to the manufacturer's instructions (ABCAM, USA). Plasmatic cortisol levels will be analyzed in the laboratory of the University Hospital of the Pontificia Universidad Católica de Chile.

Light and noise measurements: A sonometer (Cirrus CR:172B, Cirrus Research, UK) and a luxometer will be placed near the patient's head. Ambient sound will be measured continuously and illuminance will be measured every 30 minutes until ICU discharge. These data will be registered and analyzed later by light and sound experts.

ICU Delirium: The presence of delirium will be evaluated once daily using the Confusion Assessment Method for the ICU (CAM-ICU)(72) starting immediately after randomization and until ICU discharge. Prevalence of delirium will be defined as the presence of delirium (positive CAM-ICU screening) on at least 1 day during ICU stay. Duration of delirium will be defined as total days of delirium during ICU stay.

Long term Neuropsychological impairment

- Beck´s Depression Inventory (BDI-II)(73): this instrument screens for depression using criteria consistent with the Diagnostic and Statistical Manual of Mental Disorders-Fourth Edition. Higher scores (range, 0-63) indicate more depressive symptoms. Based on testing in psychiatric outpatients, depression symptoms severity is classified as minimal score (score 0-13), mild (score 14-19), moderate (score 20-28) and severe (score 29-63). The BDI-II will be performed at hospital discharge and 6 months after ICU discharge.
- Repeatable Battery for the Assessment of Neuropsychological Status (RBANS)(74-76): The RBANS is a comprehensive and validated neuropsychometric battery for the evaluation of global cognition, including individual do mains of immediate and delayed memory, attention, visuospatial construction and language. The RBANS will be performed at hospital discharge and 6 months after ICU discharge.

g. Sample size and statistical analysis

According to a previous study, which measured sleep with PSG (63), and other studies, which have described sleep architecture in the ICU (3-7), we estimate that the N3 stage time in our study population will be 7 minutes with a SD of 9 minutes. We considered that a 50% improvement in N3 stage time would be both, clinically relevant and realistici. With a power of 80% and with alpha of 5%, we calculated that a sample size of 56 patients is required to demonstrate statistically significant differences.

General statistical analysis will be performed according to intention to treat. Baseline characteristics will be reported using mean values ± standard deviation (SD), or median (p25-75) and proportions where

appropriate. The use of parametric vs non-parametric tests will be decided according to data distribution and the fulfillment or not of normality assumptions. Specific statistical analysis for comparisons between groups will be performed by Student´s-t test or Mann-Whitney U-test for continuous variables, and by chi-square for proportions. Changes along time will be analyzed by one-way (repeated-measures) analysis of variance (ANOVA) or Friedman test. Interactions between groups and time we will assessed with two-way repeated-measures ANOVA. All statistical calculations will be performed using Stata Statistical Software, Release 15.1 (College Station, TX). A probability value (p-value) of less than 0.05 will be considered as statistically significant.

IV. Work plan

We request funding for a 4-year study because during the first year of study we will implement the intervention in the ICU. We will recruit 56 patients, and considering the admission database of our ICU (in our ICU 1600 patients are admitted per year), we estimate that 2 - 3 patients could be included per month. We require at least 24 months of continuous screening to meet this target and complete the follow-up, starting inclusion by the 8th month of the project, and finishing the 6th month of the four year.

**Aim or task**	Year1 Sem1	Year1 Sem2	Year2 Sem1	Year2 Sem2	Year3 Sem1	Year3 Sem2	Year4 Sem1	Year4 Sem2
Purchase of equipment and material	X	X	X					
Implementation of intervention (modifications of the 4 ICU rooms), developtment of protocol for the nocturnal patients care activities, neasurements and readjustments	X	X	X					
Pilot		X						
Recruitmen and follow up of patient for clinical study		X	X	X	X	X	X	
Analysis of collected variables			X	X	X	X	X	X
Presentation of preliminary data at congress				X		X		X
Preparation of final report							X	X
Preparation and submission of manuscript							X	X

V. Information in support of team capacity and experience to conduct the project

Our Department (Departamento de Medicina Intensiva) has a robust track record in research. Therefore, we have highly trained and skilled professionals in the development of clinical studies, and we have conducted extensive research in critically ill patients.

The principal investigator (LA) has a long-time record of research in this group working in critical illness. She is expert in critical care and epidemiology and has collaborated as co-researcher and staff in various research projects in the last 10 years (FONDECYT grants 1100610, 1130200, 1161556, 1170043, among others). In 2015 she was co-founder of the LATIN AMERICAN INTENSIVE CARE NETWORK (LIVEN)[www.redliven.org], a research network that already published three important papers and with several other ongoing studies.

Co-investigators will address specific technical aspects. Pablo Brockmann (MD, PhD) is a sleep specialist and has an active line of research in sleep, neurocognitive disorders, sleep spindles, parasomnias and asthma. Douglas Leonard (Electric engineer) is a leading lighting specialist who has directed important lighting projects and numerous research projects in the area. Rodrigo Cádiz (Electric engineer, PhD) is a leading specialist in sound synthesis, digital signal processing. Paula Repetto (Ps., PhD) is specialist in long-term cognitive outcomes. The solid research trajectory of this interdisiplinary team ensures the feasibility of the project, and provides a solid theoretical bases for the design of the present project.

Finally, we also have the collaboration and support of international expert professors in sleep in critical patient and long-term cognitive outcomes after critical illness (Prof. Elizabeth Wilcox, Toronto Western Hospital MSNICU, Toronto, Canadá).
