# Supplementary figures and images for: Improve sleep in critically ill patients: Study protocol for a randomized controlled trial for a multi-component intervention of environment control in the ICU
Source: PLoS One. 2023 May 25;18(5):e0286180. doi: 10.1371/journal.pone.0286180 (PMC10212109; doi:10.1371/journal.pone.0286180)

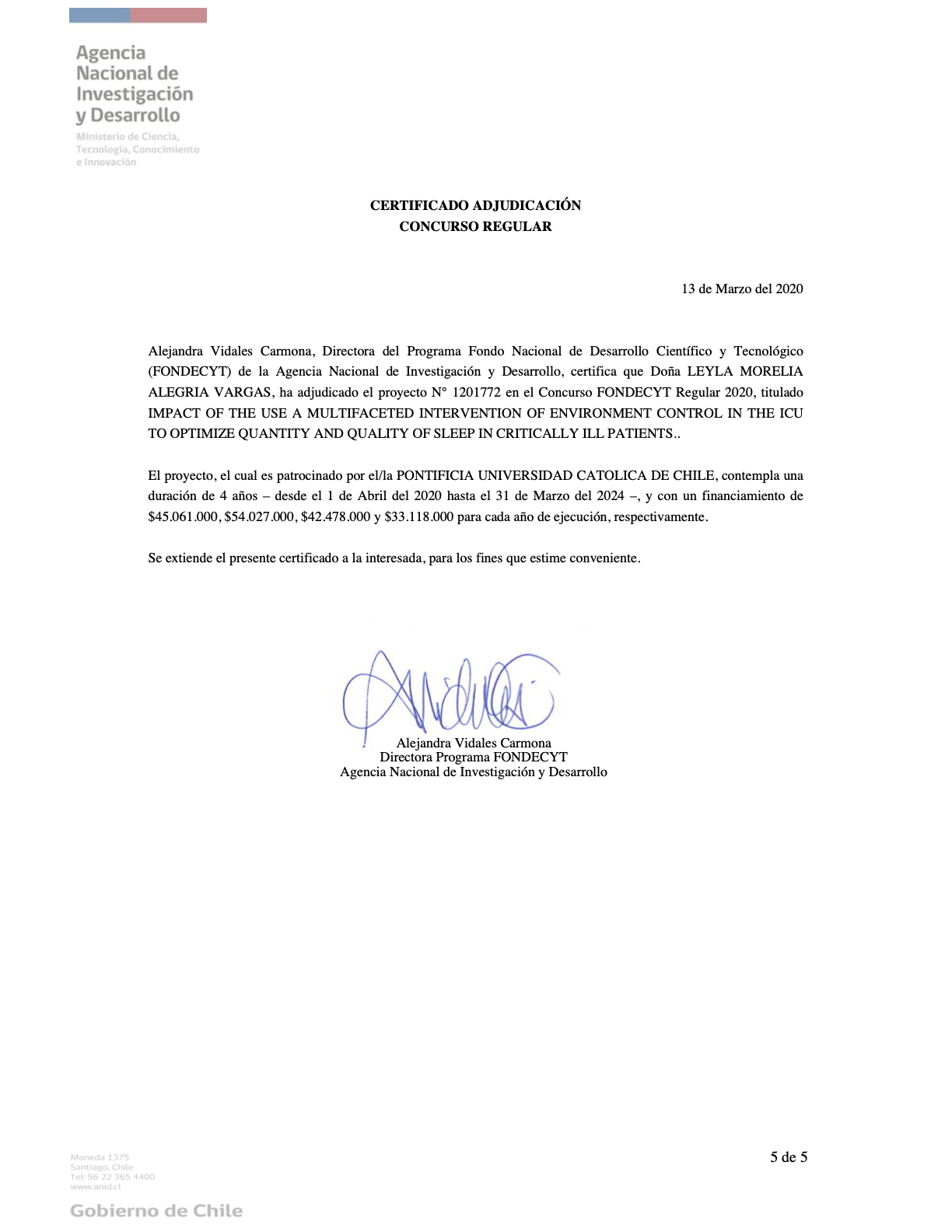

Supplement: S3 Appendix — (PNG) [file pone.0286180.s004.png]
